# Impacts of Premature Atrial Contractions and Biochemical Markers Early After Cryoballoon Versus Radiofrequency Ablation on the Late Recurrence of Atrial Fibrillation

**DOI:** 10.19102/icrm.2025.16043

**Published:** 2025-04-15

**Authors:** Kenichi Sasaki, Daisuke Togashi, Akira Kasagawa, Ikutaro Nakajima, Takumi Higuma, Tomoo Harada, Yoshihiro J. Akashi

**Affiliations:** 1 Department of Cardiology, St. Marianna University School of Medicine, Kawasaki, Japan; 2 Department of Cardiology, Kawasaki Municipal Tama Hospital, Kawasaki, Japan

**Keywords:** Ablation, atrial fibrillation, Holter, premature atrial contraction, recurrence

## Abstract

We sought to clarify the impacts of premature atrial contractions (PACs) and biochemical markers early after cryoballoon (CB) versus radiofrequency (RF) ablation for atrial fibrillation (AF) on the late recurrence of AF (LRAF). The study population included 138 patients who underwent first-time ablation for paroxysmal AF with CB (*n* = 69) or RF (*n* = 69). We compared the levels of the PAC burden on Holter monitoring, myocardial-bound creatine kinase (CK-MB), troponin T (TnT), and C-reactive protein (CRP) the day after ablation, and we assessed the incidence of LRAF, which was defined as AF after a 3-month blanking period. The postprocedural PAC burden was not significantly different between the CB and RF groups (*P* = .35), whereas the CK-MB and CRP levels were significantly higher in the CB group (both *P* < .01); the TnT levels of the groups were similar (*P* = .63). Among these, only a higher PAC burden was significantly associated with LRAF in both the CB (top quartile [≥2.16%]: 58% vs. others: 17%; log-rank *P* = .01) and RF (top quartile [≥3.05%]: 36% vs. others: 9%; log-rank *P* < .01) groups. A Cox regression analysis revealed two significant predictors of LRAF: in-hospital recurrence (CB group: hazard ratio [HR], 3.55 [1.67–11.80]; *P* = .04; RF group: HR, 7.55 [1.67–34.20]; *P* = .01) and a higher postprocedural PAC burden (CB: HR, 1.54 [1.06–2.22]; *P* = .02; RF: HR, 1.90 [1.16–3.35]; *P* = .01). In conclusion, irrespective of the ablation modality, the next-day PAC burden (but not the biochemical markers examined herein) is useful for predicting LRAF. Early AF recurrence should be considered a future risk even at the beginning of the blanking period.

## Introduction

Frequent premature atrial contractions (PACs) are associated with not only the development of atrial fibrillation (AF) but also the recurrence of AF after catheter ablation. Several research groups investigated the behavior of PACs on Holter monitoring immediately after AF ablation and revealed a relationship between the extent of the PAC burden and recurrent AF.^1–3^ Other studies have focused on serum biomarkers reflecting myocardial cell damage or inflammation, and they demonstrated that AF ablation leads to transient elevations in myocardial-bound creatine kinase (CK-MB), troponin T (TnT), and C-reactive protein (CRP) after the procedure.^4–9^ These biochemical changes appear to result from a histopathological process in which thermal damage causes protein denaturation and disruption of the myocardial cell membrane, leading to myocardial necrosis and subsequent inflammatory infiltration.^10^

However, these observations were primarily based on findings from radiofrequency (RF) ablation studies. The data on cryoballoon (CB) ablation are limited. We thus conducted the present study to investigate (1) the postprocedural behavior of PACs evaluated by Holter monitoring, (2) CK-MB and TnT as markers of myocardial injury, and (3) CRP as a marker of inflammation on the day after CB ablation for paroxysmal AF, and we compared the results with those of patients who underwent RF ablation. We also examined the association between these postprocedural parameters and long-term recurrent AF after ablation.

## Methods

### Study population

We prospectively recruited consecutive patients who underwent CB or RF ablation for paroxysmal AF for the first time between December 2021 and August 2023 at our institutions. Study exclusion criteria were as follows: (1) prior or periprocedural pacemaker implantation, which could influence the occurrence of AF episodes due to ventricular pacing, and (2) any ablation procedures beyond pulmonary vein (PV) isolation (PVI) and cavotricuspid isthmus (CTI) ablation. The final study population consisted of 138 patients who underwent CB ablation (n = 69) or RF ablation (n = 69). The study protocol was approved by the St. Marianna University Ethics Committee (approval no. 5479), and all patients provided their written informed consent regarding the performance of the ablation procedure and the use of their clinical data for research purposes.

### Ablation procedure

All anti-arrhythmic drugs were discontinued for at least five half-lives prior to ablation. The choice of the ablation modality was not randomized but was left to the operators’ discretion. All procedures were performed with the patient under general anesthesia with remifentanil and propofol. After a transseptal puncture, intravenous heparin was administered to maintain an activated clotting time of 300–350 s.

For the CB ablation cases, a second-generation 28-mm balloon (Arctic Front Advance; Medtronic, Minneapolis, MN, USA) was inserted into the left atrium (LA), and cryotherapy was applied to the ostium of the left and right superior/inferior PVs. The standard freezing time was 180 s; however, this timing was also left to the discretion of the operator, depending on the time to isolation, the temperature-lowering speed, and/or the nadir. To avoid phrenic nerve injury, phrenic nerve pacing was performed during the freezing of the right PV via a quadripolar catheter. The capture of phrenic nerves was documented via a manual confirmation of diaphragmatic movement and compound motor action potentials. Freezing was immediately stopped in the cases of cessation or weakening of the diaphragmatic movement.

In the RF ablation cases, extensive encircling PVI and electroanatomic mapping combined with image integration were performed for all patients. Circumferential ablation lines were created around the ipsilateral PVs with the use of a 3.5-mm-tip irrigation catheter (SmartTouch SF; Biosense Webster, Diamond Bar, CA, USA). RF energy was delivered with a power of up to 35–40 W and was limited to 20–30 W near the esophagus.

In both the CB and RF groups, the endpoint of ablation was the elimination of all PV potentials. If there were electrical gaps or reconnections in the ablation lines after the initial procedure, an additional “touch-up” ablation was conducted using an irrigation catheter in both groups. After PVI, CTI ablation was performed using RF in all study populations. No further ablation beyond PVI and CTI ablation was allowed during the index procedure.

### Holter monitoring

The patients routinely underwent 24-h Holter monitoring (FM-180; Fukuda Denshi, Tokyo, Japan) 24 h after the ablation procedure. The Holter monitoring data were automatically interpreted by a Holter analyzer (SCM-8000; Fukuda Denshi), manually analyzed by experienced cardiac technicians, and reviewed by a responsible investigating cardiologist. A PAC was defined as any supraventricular complex that occurred earlier than the previous R–R interval and which had a P-wave that differed from the sinus rhythm.

We extracted the following parameters from the records: (1) the PAC burden (%), calculated by dividing the number of PACs by total heartbeats and multiplying by 100; (2) the number of PAC runs, which was defined as more than two consecutive PACs; (3) the longest PAC run; and (4) the PAC coupling interval. To estimate the PAC burden, we used the percentage of PACs instead of the total number of PACs because the number of PACs depended on the entire recording time, which exhibited some inter-individual variation. The PAC burden was further categorized by the distribution in quartiles. Each PAC coupling interval, which was calculated as a percentage of each preceding R–R interval, was automatically measured using SCM-8000 software. AF was defined as either irregular supraventricular activity without monomorphic P-waves or irregular supraventricular activity with F-waves lasting ≥30 s. If episodes of AF were detected during a patient’s Holter recording, the periods of AF were manually excluded, and the number of PACs in the remaining period was analyzed.

### Laboratory examinations

Blood samples for the measurement of CK-MB, TnT, and high-sensitivity CRP were collected approximately 24 h after the CB or RF procedure. CK-MB levels of <3.6 ng/mL, TnT levels of <0.014 ng/mL, and CRP levels of <0.14 mg/dL were considered normal by our laboratory. If the measurement was below the limit of quantification, the lowest value was adopted. The data for each biomarker were further categorized based on their distribution into quartiles.

### Follow-up and study endpoints

All patients were hospitalized for at least 2 days after ablation under continuous electrocardiogram (ECG) monitoring. After discharge, the patients were scheduled for follow-up visits at 1, 3, 6, and 12 months and then every 6 months thereafter. Holter monitoring was recommended as a regular check-up or when patients reported symptoms suggestive of recurrence at any visit. All patients were also encouraged to check their pulse rate and heart rhythm every day and to visit an outpatient clinic if they experienced irregular pulses.

In-hospital AF recurrence (very early recurrence of AF [VERAF]) was defined as documented AF or atrial tachyarrhythmia lasting ≥30 s on the patient’s planned Holter monitoring or the ECG monitoring during hospitalization. Late recurrence of AF (LRAF), which was our study’s primary endpoint, was defined as AF or atrial tachyarrhythmia lasting ≥30 s after a 3-month blanking period documented in at least one of the following ECG recordings: 12-lead ECG, 24-h or 2-week Holter monitoring, or a wearable ECG monitoring device.

### Statistical analysis

Continuous variables are presented as mean ± standard deviation or median (interquartile range) values and were compared using Student’s *t* test or Wilcoxon’s rank sum test, depending on the distribution of the variables. Categorical values are presented as counts and percentages and were compared using Fisher’s exact test. Logarithmic transformations were applied to certain log-normally distributed variables (CK-MB, CRP, the PAC burden, the number of PAC runs, and the longest PAC run) for regression analyses. The correlations between variables were analyzed using Pearson’s correlation tests.

Univariable logistic regression models were assessed to identify the postprocedural ECG or biochemical parameters associated with VERAF. Univariate Cox proportional regression analyses were performed to assess the association of the PAC burden 1 day after ablation and the known predictors of recurrence (ie, the LA diameter and VERAF) with LRAF. Two-sided *P* values of <.05 were considered significant. All statistical analyses were performed using JMP^®^14 (SAS Institute, Cary, NC, USA).

## Results

### Patient characteristics

The baseline characteristics of the CB and RF groups are summarized in **Table 1**. The two groups did not differ significantly except for the CB group’s significantly higher estimated glomerular filtration rates (eGFRs) (68 ± 16 vs. 62 ± 19 mL/min/1.73 m^2^; *P* = .03) and significantly lower levels of N-terminal pro-brain natriuretic peptide (64 [33–142] vs. 158 [67–387] pg/mL; *P* < .01).

**Table 1:** Patient Characteristics

	CB Group n = 69	RF Group n = 69	*P* Value
Age (years)	66 ± 10	65 ± 9	.63
Women, n (%)	23 (33)	18 (26)	.46
Body mass index (kg/m^2^)	24 ± 4	24 ± 4	.75
Time since first AF episode (months)	6 (3–16)	5 (3–26)	.78
Hypertension, n (%)	35 (51)	31 (45)	.61
Diabetes mellitus, n (%)	11 (16)	14 (20)	.66
History of CAD, n (%)	8 (12)	7 (10)	1.00
History of HF, n (%)	1 (1)	5 (7)	.21
eGFR (mL/min/1.73 m^2^)	68 ± 16	62 ± 19	.03
NT-proBNP (pg/mL)	64 (33–142)	158 (67–387)	<.01
Left atrial diameter (mm)	36 ± 5	34 ± 11	.65
LVEF (%)	63 ± 5	62 ± 6	.35

*Abbreviations:* AF, atrial fibrillation; CAD, coronary artery disease; CB, cryoballoon; eGFR, estimated glomerular filtration rate; HF, heart failure; LVEF, left ventricular ejection fraction; NT-proBNP, N-terminal pro-brain natriuretic peptide; RF, radiofrequency.

### Procedural characteristics

The details of the procedural characteristics are presented in **Table 2**. Compared to the RF group, the CB group had significantly shorter procedural times (170 ± 30 vs. 189 ± 35 min; *P* = .02) and RF delivery times (8 ± 5 vs. 36 ± 12 min; *P* < .01). The fluoroscopy time, complication rate, and VERAF rate did not differ significantly between the groups (*P* = .77, 1.00,. 36, respectively).

**Table 2:** Procedural Data

	CB Group n = 69	RF Group n = 69	*P* Value
Procedural time (min)	170 ± 30	189 ± 35	.02
Fluoroscopy time (min)	12 ± 3	12 ± 4	.77
RF delivery time (min)	8 ± 5^a^	36 ± 12	<.01
Occlusion time with CB (s)			
LSPV	254 ± 86	—	
LIPV	191 ± 47	—	
RSPV	189 ± 37	—	
RIPV	205 ± 66	—	
First-pass isolation with RF, n (%)			
LPV	—	48 (70)	
RPV	—	59 (86)	
Complications	1 (0)	0 (0)	1.00
Phrenic nerve palsy, n (%)	1 (0)	0 (0)	
Very early recurrence of AF, n (%)	14 (20)	9 (13)	.36

*Abbreviations:* AF, atrial fibrillation; CB, cryoballoon; CTI, cavotricuspid isthmus; LIPV, left inferior pulmonary vein; LPV, left pulmonary vein; LSPV, left superior pulmonary vein; RF, radiofrequency; RIPV, right inferior pulmonary vein; RPV, right pulmonary vein; RSPV, right superior pulmonary vein. ^a^RF delivery time in the CB group includes the time required for touch-up and CTI ablations using RF.

### Postprocedural levels of premature atrial contractions and biochemical markers

**Table 3** presents the results of our comparisons between the CB and RF groups regarding PAC burden on Holter monitoring and the levels of CK-MB, TnT, and CRP 1 day after AF ablation. PAC burden did not differ to a significant extent between the two groups (*P* = .35), but the levels of CK-MB and CRP on the day after ablation were significantly higher in the CB group (both *P* < .01). The TnT level was similar between the two groups (*P* = .63).

**Table 3:** 24-h Holter Monitoring and Laboratory Data 1 Day After Ablation

	CB Group n = 69	RF Group n = 69	*P* Value
24-h Holter monitoring findings			
Monitoring duration (h)	23.7 ± 0.9	23.5 ± 1.1	.35
Total number of heartbeats/record	104,479 ± 19,080	106,518 ± 20,722	.55
Mean heart rate (beats/min)	79 ± 11	79 ± 11	.90
Number of PACs (beats/record)	687 (376–2037)	636 (212–3210)	.34
PAC burden (%)	0.69 (0.36–2.16)	0.63 (0.21–3.05)	.35
Biochemical markers			
CK-MB (ng/mL)	27.3 (20.1–41.7)	5.5 (4.2–7.8)	<.01
Troponin T (ng/mL)	1.62 ± 0.42	1.66 ± 0.55	.63
CRP (mg/dL)	0.66 (0.41–1.11)	27 (0.26–0.84)	<.01

*Abbreviations:* CB, cryoballoon; CK-MB, myocardial-bound creatine kinase; CRP, C-reactive protein; PAC, premature atrial contraction; RF, radiofrequency.

### Postprocedural parameters and very early recurrence of atrial fibrillation

Early AF recurrence during hospitalization occurred in 23 (17%) patients (14 in the CB group vs. 9 in the RF group; *P* = .36) **(Table 2)**. The results of the univariate logistic regression analyses to assess the association of postprocedural parameters with VERAF are listed in **Table 4**. In both ablation groups, only the ln next-day PAC burden was identified as a predictive factor (CB group: odds ratio [OR], 1.79; 95% confidence interval [CI], 1.14–2.80; *P* = .01; RF group: OR, 3.15; 95% CI, 1.49–6.66; *P* < .01). None of the biochemical markers’ data predicted VERAF.

**Table 4:** Univariate Logistic Regression Model of Variables Associated with In-hospital Recurrence of Atrial Fibrillation in the Cryoballoon and Radiofrequency Groups

	Odds Ratio (95% CI)	*P* Value
CB group		
Next-day PAC burden^a^	1.79 (1.14–2.80)	.01
Next-day CK-MB^a^	3.16 (0.86–11.62)	.08
Next-day troponin T	0.56 (0.13–2.39)	.44
Next-day CRP^a^	1.20 (0.61–2.39)	.60
RF group		
Next-day PAC burden^a^	3.15 (1.49–6.66)	<.01
Next-day CK-MB^a^	1.40 (0.38–5.15)	.61
Next-day troponin T	1.94 (0.51–7.46)	.33
Next-day CRP^a^	1.17 (0.51–2.70)	.72

*Abbreviations:* AF, atrial fibrillation; CB, cryoballoon; CI, confidence interval; CK-MB, myocardial-bound creatine kinase; CRP, C-reactive protein; PAC, premature atrial contraction; RF, radiofrequency. ^a^Evaluated as log-transformed values.

### Impacts of postprocedural parameters on late recurrence of atrial fibrillation

**Figure 1:**
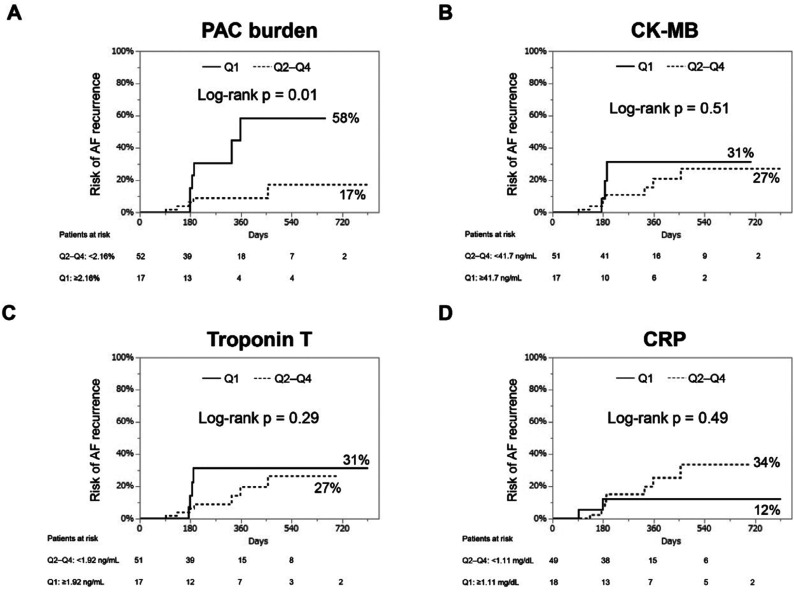
Kaplan–Meier curves of the cumulative risk of late atrial fibrillation recurrence in patients who underwent cryoballoon ablation (n = 69). We stratified the patients into the top quartile (Q1) and the other quartiles (Q2–Q4) based on the levels of premature atrial contraction burden **(A)**, myocardial-bound creatine kinase **(B)**, troponin T **(C)**, and C-reactive protein **(D)**. *Abbreviations:* AF, atrial fibrillation; CK-MB, myocardial-bound creatine kinase; CRP, C-reactive protein; PAC, premature atrial contraction.

**Figure 2:**
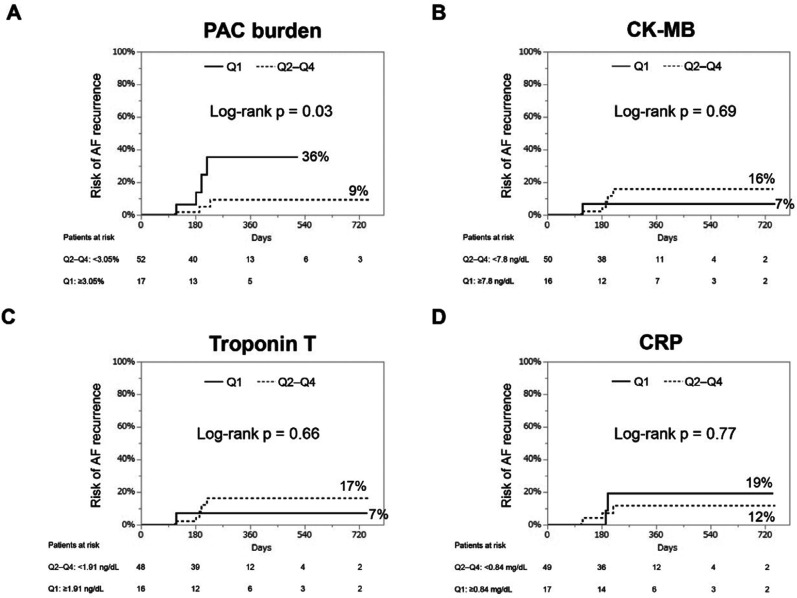
Kaplan–Meier curves of cumulative risk of late atrial fibrillation recurrence in patients who underwent radiofrequency ablation (n = 69). We stratified these patients into the top quartile (Q1) and the other quartiles (Q2–Q4) based on the levels of premature atrial contraction burden **(A)**, myocardial-bound creatine kinase **(B)**, troponin T **(C)**, and C-reactive protein **(D)**. *Abbreviations:* AF, atrial fibrillation; CK-MB, myocardial-bound creatine kinase; CRP, C-reactive protein; PAC, premature atrial contraction.

During a median follow-up period of 6 (interquartile range, 5–12) months, 18 (13%) patients experienced LRAF. Kaplan–Meier curves for the cumulative risk of LRAF stratified by next-day levels of the PAC burden, CK-MB, TnT, and CRP in quartiles are illustrated in **Figure 1** (CB group) and **Figure 2** (RF group). In both groups, patients with a postprocedural PAC burden in the top quartile (≥2.16% in the CB group and ≥3.05% in the RF group) had an increased risk of LRAF compared to patients in the other quartiles (CB group: top quartile 58% vs. others 17%; log-rank *P* = .01; RF group: top quartile 36% vs. others 9%; log-rank *P* < .01). However, patients with CK-MB, TnT, or CRP values in the top quartile did not show higher incidences of LRAF.

The univariate Cox regression analyses **(Table 5)** revealed the following as significant predictors of LRAF: (1) the ln next-day PAC burden with a hazard ratio (HR) of 1.54 (95% CI, 1.06–2.22; *P* = .02) in the CB group and 1.90 (95% CI, 1.16–3.35; *P* = .01) in the RF group and (2) VERAF with an HR of 3.55 (95% CI, 1.67–11.80; *P* = .04) in the CB group and 7.55 (95% CI, 1.67–34.20; *P* = .01) in the RF group.

**Table 5:** Univariate Cox Proportional Hazards Regression Model of Variables Associated with Long-term Recurrence of Atrial Fibrillation in the Cryoballoon and Radiofrequency Groups

	Hazard Ratio (95% CI)	*P* Value
CB group		
Next-day PAC burden^a^	1.54 (1.06–2.22)	.02
Left atrial diameter (per 1-mm increment)	1.12 (0.97–1.31)	.13
In-hospital recurrence of AF	3.55 (1.67–11.80)	.04
RF group		
Next-day PAC burden^a^	1.90 (1.16–3.35)	.01
Left atrial diameter (per 1-mm increment)	1.08 (0.96–1.23)	.21
In-hospital recurrence of AF	7.55 (1.67–34.20)	.01

*Abbreviations:* AF, atrial fibrillation; CB, cryoballoon; CI, confidence interval; PAC, premature atrial contraction; RF, radiofrequency. ^a^Evaluated as a log-transformed value.

## Discussion

### Main findings

This study clarified (1) the differences in the postoperative PAC burden and biochemical markers between patients who underwent CB ablation and patients who underwent RF ablation and (2) the predictors of VERAF and LRAF in each group. Various reports have discussed the behavior and importance of PACs after AF ablation,^1–3,11–13^ but, to the best of our knowledge, no study to date has compared postoperative PACs between CB and RF ablation or the clinical impacts of these procedures. Our analyses revealed that, in patients undergoing CB or RF ablation for paroxysmal AF, (1) there were no significant between-group differences with respect to the PAC burden on Holter monitoring and the level of TnT, but significantly higher levels of CK-MB and CRP were observed in the CB group the day after ablation; (2) patients with a higher PAC burden had a higher incidence of LRAF; and (3) a higher PAC burden and VERAF were significant predictors of LRAF after CB ablation and after RF ablation.

### Myocardial injury in cryoballoon versus radiofrequency ablation

Several investigations have compared serum biomarkers of myocardial injury between CB and RF energies after AF ablation. Some of these studies reported that CB caused more myocardial injury than RF,^6,7,9^ while others described a comparable increase in CB and RF ablations^5,8^ or, contrarily, more myocardial damage in RF.^4^ Our present analyses demonstrated that the CB group had higher levels of CK-MB and CRP than the RF group, despite having similar TnT levels.

There are several possible explanations for this discrepancy. First, there are between-study differences in (1) the patient background; (2) the technical background among operators or institutes, such as the method of touch-up ablation, CB freezing time, applied RF power, or type of ablation catheter; (3) the blood sampling timing; and (4) the laboratory assays used to measure biomarkers. Regarding the patient background, a lower eGFR can result in reduced clearance of myocardial biomarkers, leading to their elevation. On the contrary, the CB group in the present study had a higher eGFR than the RF group due to the operators’ selection bias precluding CB ablation requiring more contrast media for patients with low eGFRs. The most likely reason for the higher levels of CK-MB in our CB ablation group is the thermal instability of CK-MB. An in vitro study by Wojcik et al. showed that CK-MB was inactivated by heating, and a substantial drop was observed at 40°C, while troponin I was stable.^14^ It can thus be speculated that this phenomenon was responsible for the higher CK-MB levels after CB ablation and the lower CK-MB levels after RF ablation, regardless of the comparable troponin levels.

Regarding the association between myocardial injury and recurrent AF, Lim et al. reported that higher TnT levels were a predictor of AF recurrence within the first 3 days after RF ablation,^15^ while Aksu et al. reported that lower troponin I levels increased the risk of mid-term recurrence after CB ablation for AF.^16^ It seems reasonable that greater myocardial damage would indicate more effective lesion formation and correlate with a better prognosis. However, our analysis did not show any association between TnT levels and AF recurrence. If this hypothesis holds true, increased myocardial damage would lead to more inflammation and a higher PAC burden, which contradicts the finding that a lower PAC burden is associated with a better prognosis. Therefore, it is essential to quantify the lesion size resulting from ablation to accurately assess the relationship between this biomarker and post-ablation outcomes.

### Inflammatory response after cryoballoon versus radiofrequency ablation

As is the case with myocardial injury, the inflammatory response after CB ablation and RF ablation remains controversial. Hayazaki et al. and Miyazaki et al. reported similar CRP levels,^6,7^ whereas Yano et al. observed higher CRP levels in their RF group.^9^ In contrast, our CB group had higher CRP levels than the RF group. We observed a weak positive correlation between CK-MB and CRP (*r* = 0.20; *P* = .02), which was consistent with the report by Lim et al.^15^ This could partly explain the higher CRP levels in our CB group.

It has been pointed out that the elevation of CRP during the acute phase is a predictor of early recurrence post-PVI.^6,15^ Referring to the usefulness of corticosteroids in preventing acute recurrence after AF ablation,^17^ we speculate that inflammation could theoretically be associated with early recurrence, although our present analyses did not reveal a relationship between VERAF and patients’ CRP levels. When discussing inflammation after ablation, the time course of CRP elevation should also be considered. The CRP level peaks 2–3 days later than other biochemical markers after ablation.^6,15^ The single measurement after ablation in our method could not accurately evaluate the CRP level.

### Clinical impact of the premature atrial contraction burden early after cryoballoon versus radiofrequency ablation

Our findings demonstrated that the PAC burden on Holter monitoring the day after AF ablation did not differ significantly between the CB and RF groups, and we observed that a greater PAC burden was a significant risk factor for VERAF and LRAF in both groups. Numerous studies have described an association between a high PAC burden and AF recurrence after ablation, observed mainly in patients undergoing RF ablation. We also observed this association in patients who underwent CB ablation.

**Figure 3:**
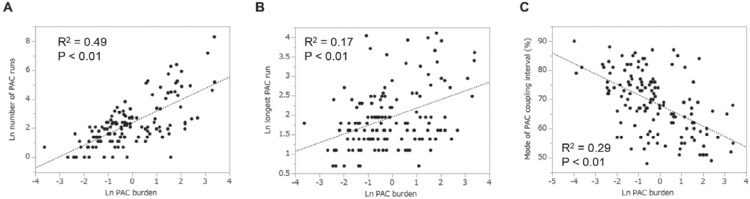
The correlations between the premature atrial contraction (PAC) burden and other PAC-related parameters, including the number of PAC runs **(A)**, the longest PAC run **(B)**, and the mode of PAC coupling interval **(C)**. The values of PAC burden, the number of PAC runs, and the longest PAC run were logarithmically transformed before plotting.

In addition, several studies have described the usefulness of PAC-related parameters other than the PAC burden on Holter monitoring, including the longest PAC run or the PAC coupling interval, as a predictor of LRAF.^1,13^ As displayed in **Figure 3**, the PAC burden and other representative PAC-related parameters are correlated to some extent; it is therefore reasonable that the PAC burden is used as a representative marker of postprocedural PAC behavior.

We also investigated PAC-related parameters 1 month after the index ablation in a subset of the study population. The results demonstrated that the levels of PAC-related parameters, including the PAC burden, the number of PAC runs, and the longest PAC run, were decreased 1 month after the procedure in patients without LRAF **(Table 6)**. We thus speculate that the increased PAC burden in this cohort would be a transient phenomenon due to inflammation induced by myocardial injury or provoked autonomic nervous system post-ablation. Patients with LRAF still had higher PAC burdens 1 month after ablation, and we thus consider that the next-month PAC burden could be more useful to predict LRAF. Regarding the postprocedural PAC coupling interval, patients with LRAF had a shorter PAC coupling interval than those without through 1-month follow-up. In addition, the mode of PAC coupling interval did not differ between 1 day and 1 month after ablation in both groups. Considering the previously reported finding that PACs with a short coupling interval derive from the PVs,^18^ frequent PACs early after PVI are caused by reconnected PV–LA conduction in patients with LRAF, whereas, in patients without LRAF, factors unrelated to PV–LA connection, such as inflammation, are more likely to contribute.

**Table 6:** Comparison of Parameters Related to Premature Atrial Contractions 1 Day and 1 Month After Ablation Between Patients with and Without Late Atrial Fibrillation Recurrence

	LRAF (−)			LRAF (+)		
	1 Day	1 Month	*P* Value	1 Day	1 Month	*P* Value
PAC burden (%)	0.56 (0.23–1.86)	0.17 (0.05–0.60)	<.01	3.75 (0.57–6.77)	2.82 (0.56–5.04)	.43
No. of PAC runs (times/record)	6 (2–13)	3 (1–10)	<.01	25 (8–210)	14 (5–83)	.44
Longest PAC run (beats)	6 (4–10)	4 (3–8)	.04	7 (5–25)	10 (8–43)	.82
Mode of PAC coupling interval (%)	70 ± 10	70 ± 11	.36	63 ± 10	66 ± 14	.35

*Abbreviations:* LRAF, late recurrence of atrial fibrillation; PAC, premature atrial contraction.

### Clinical impact of in-hospital atrial fibrillation recurrence after cryoballoon versus radiofrequency ablation

The present study demonstrated that not only a higher PAC burden but also VERAF were significant predictors of LRAF. Although this finding was observed mainly after RF ablation, it was common in both the CB and RF groups. On the contrary, there is a well-established consensus that early recurrence is not associated with long-term prognosis, on which the concept of the 3-month blanking period is based. What is this discrepancy derived from? One of the possible reasons is the difference in the timing of AF recurrence. The majority of earlier studies defined the recurrence of AF within 90 days after ablation as early recurrence, whereas, in the present study, early AF recurrence was limited to in-hospital recurrences, ie, within 2 days post-ablation. We selected this timing because continuous ECG monitoring was available during hospitalization and can prevent an underestimation of recurrent AF. Moreover, we focused on laboratory data obtained immediately after AF ablation.

Nevertheless, several studies revealed VERAF to be a significant and strong predictor of LRAF,^19–21^ and our present results confirm this finding and question the concept of a blanking period after AF ablation. A large LA diameter is an established predictor of recurrent AF after PVI, but we could not evaluate this factor in the present analyses because both the CB and RF groups had a relatively small LA (mean diameter < 40 mm).

### Clinical implications

Some biochemical markers after ablation differed between the CB and RF groups; however, these differences did not correlate with post-ablation prognosis. In contrast, a higher PAC burden early after ablation was associated with both early and late recurrence. Evaluating PAC behavior with 24-h Holter monitoring during hospitalization is relatively feasible, allowing for the early identification of patients at high risk of AF recurrence who may benefit from closer observation, such as repeated 24-h or 2-week Holter monitoring. While a 3-month blanking period after AF ablation is widely accepted, recent clinical data suggest less duration.^22,23^ Thus, we believe that screening patients with a higher PAC burden through Holter monitoring the day after ablation is not necessarily an overestimation.

### Limitations

This study has several limitations to address. First, the sample size was relatively small (n = 138), which reduced the statistical ability of our results to identify predictors of LRAF. Second, the lack of data on the size of the lesion created by the two energy sources prevented an accurate assessment of the magnitude of the injured tissue. Third, precise data on medications that could affect PACs or the recurrence of AF were not available; however, anti-arrhythmic drugs were not prescribed after the procedure in most of the patients. Fourth, the number of PACs on Holter monitoring could have been underestimated due to the presence of blocked PACs. Lastly, underestimating asymptomatic AF is the most significant limitation of our study. While we addressed this by encouraging patients to perform daily pulse checks, this method is not always a reliable indicator.

## Conclusions

A higher PAC burden on the day after ablation for paroxysmal AF was a significant predictor of not only VERAF but also LRAF both in the CB and RF groups. The biochemical markers related to myocardial injury or inflammation were not predictive. An early recurrence of AF should not be considered insignificant, and it should be considered a future risk even at the beginning of the blanking period.
